# A Parathyroid Adenoma: Benign Disease Presenting with Hyperparathyroid Crisis

**DOI:** 10.1155/2010/596185

**Published:** 2010-12-20

**Authors:** A. S. Tahim, J. Saunders, P. Sinha

**Affiliations:** Department of General Surgery, Princess Royal University Hospital, Farnborough Common, Orpington, Kent BR6 8ND, UK

## Abstract

Hyperparathyroid crisis is a rare manifestation of parathyroid disease. We present the case of a 53-year-old gentleman with a review of the current literature. He presented in acute renal failure with epigastric pain and vomiting. His serum-corrected calcium (CCa^2+^) was raised at 5.2 mmol/L, in addition to a massively raised parathyroid hormone (PTH) level (3957 ng/L). Ultrasound studies of the neck revealed a 2 cm well-defined mass inferoposterior to right thyroid lobe. CT scans of the neck showed a normal mediastinum and confirmed no associated lymphadenopathy. Having undergone medical resuscitation for 9 days, a neck exploration revealed a cystic mass, which was excised. Histological investigations revealed a 9.25 g, cystic parathyroid adenoma with no features of malignancy. His PTH and CCa^2+^ returned to normal postoperatively. This suspicious presentation of benign disease, including a marked elevation in PTH, highlights the challenges facing the endocrine surgeon in dealing with parathyroid disease.

## 1. Introduction

Primary hyperparathyroidism is a rare disease, with hyperparathyroid crisis being one of its unusual manifestations. Large rises in PTH levels in benign parathyroid disease are unusual and have been associated with more sinister diseases [1]. We discuss a case of a patient with a benign cystic parathyroid adenoma presenting in sinister fashion with, in particular, a massively raised serum PTH level only previously seen in malignant disease [2].

## 2. Case Report

A 53-year-old gentleman presented with a 2-week history of worsening epigastric pain, vomiting, and constipation. He reported mild confusion but no loss of consciousness. There was no history of polyuria or polydipsia. He reported longstanding gastroesophageal reflux symptoms but no other abdominal history. His medical history was negative for depression and renal calculi. There was no history of carcinoma or any radiotherapy treatment of any kind. He reported no significant family history. On examination he was pale with dry mucous membranes. His chest and abdominal examination were unremarkable. There was no evidence of any bony pain and no palpable lumps in the neck. 

**Figure 1:**
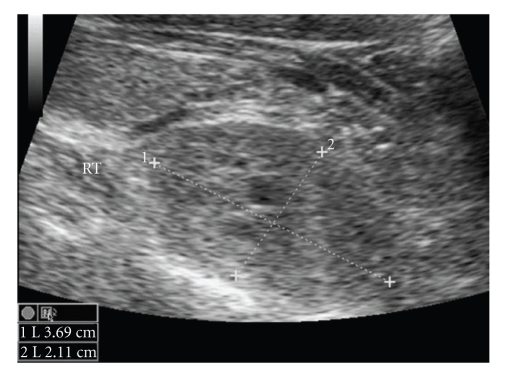
USS neck revealing 3.69 cm × 2.11 cm lesion in right side of neck.

**Figure 2:**
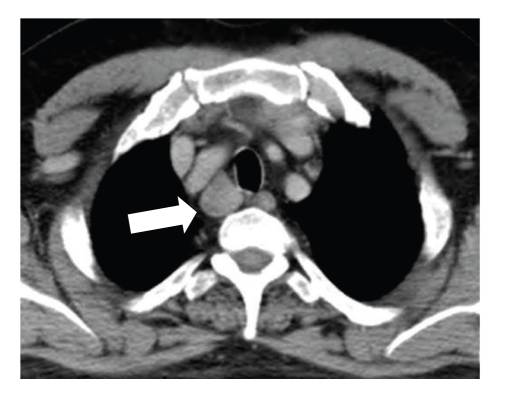
CT neck showing large right sided neck lesion.

At admission, he was tachycardic and hypotensive with an increased respiratory rate. He was in acute renal failure, with an elevated urea (19.5 mmol/L) and creatinine (272 *μ*mol/L). Liver function tests were normal. Laboratory tests revealed a markedly elevated serum-corrected calcium level (CCa^2+^) of 5.20 mmol/L (normal range 2.12–2.65) and a parathyroid hormone (PTH) level of 3957 ng/L (normal range 12–75). Vitamin D levels were normal. A myeloma screen was negative. An ECG showed that he was in normal sinus rhythm and a chest radiograph was unremarkable. Given the acute presentation and massively elevated PTH levels, the major concern was a possible malignant parathyroid lesion. An ultrasound scan of his neck revealed a 2 cm well-defined oval hypoechoic mass posterior and inferior to right thyroid lobe (Figure 1). A CT scan of the neck confirmed the 2 cm nodule as stated which abutted the right tracheal margin, with no associated lymphadenopathy and a normal mediastinum (Figure 2).

The patient underwent initial conservative management with aggressive intravenous fluid resuscitation, vitamin D replacement, intravenous loop diuretic treatment, and intravenous bisphosphonate therapy. On day 9, biochemical markers had improved (Urea 5.3, Creatinine 179), and CCa^2+^ had fallen to 2.99 mmol/L. The patient underwent a neck exploration on day 10. During the exploration, the right inferior gland was found to be large, cystic, soft, and of a brown colour. There was a no evidence of local invasion and no lymphadenopathy. As such, a diagnosis of adenoma was made. Intraoperative PTH assay is not routinely used at our unit and as such was not performed. The right inferior parathyroid gland was excised. The right superior parathyroid gland was normal, and the contralateral neck was not explored.

Histological investigations revealed a 3.5 cm × 2 cm × 1.5 cm encapsulated parathyroid adenoma weighing 9.25 g, composed of chief and oxyphil cells with cystic change. The Ki-67 proliferative index was low (1-2%). There was no local, capsular, or vascular invasion and no other features suggestive of malignancy. Given the suspicious clinical picture, the report was further confirmed at another centre.

The patient recovered well postoperatively, with PTH levels falling to 45 ng/L within a day. Subsequent recovery included a period of hypocalcaemia with a raised Alkaline Phosphatase (ALP), which resolved with calcium replacement within a month.

## 3. Discussion

Primary hyperparathyroidism is a rare disease, where the majority of patients now present asymptomatically after biochemical testing which shows a mildly raised serum CCa^2+^ and marginally elevated PTH level [3]. However, our case highlights two important aspects of parathyroid disease that the authors would like to explore further. Firstly the difficulty in diagnosis of a patient with a markedly raised PTH and secondly the difficulty faced preoperatively in the management of a patient presenting with hyperparathyroid crisis. 

Preoperative and even histological differentiation between benign or malignant pathology is often difficult, and appropriate management requires judgement at the time of surgery as malignant lesions will need more radical procedures. The features suggestive of malignant lesions at the time of surgery would include adherence due to fibrosis, local infiltration, or lymph node involvement. There has been recent work into Preoperative diagnosis based on biochemical grounds with a raised CCa^2+^ and PTH increasing the index of suspicion for malignant disease suggesting that serum PTH levels are mildly elevated in benign disease, but carcinoma causes levels up to 10 times that of normal [1]. To further stress the benefit of PTH in preoperative diagnosis, Robert et al. suggested that a PTH level <4 times the upper limit of normal excludes a malignancy [4]. Other studies support the above trend—a recent case series looking at solitary adenomas causing asymptomatic disease have derived mean values of Preoperative PTH as 186 [5] and 165 ng/L [6], while research into carcinoma show higher PTH levels of 714 [2] and 1220 ng/L [7]. This consolidates the unusual nature of a PTH level of 3957 ng/L in an adenoma with no radiological, Intraoperative, or histological evidence of malignancy. CCa^2+^ is usually no greater than 1 mmol/dl above normal ranges in benign disease compared to over 4 times the upper limit of normal in malignant lesion [1]. Tumour mass has also been shown to correlate with diagnosis. The average adenoma weighs 1 g, but the average values for carcinoma weight are approximately 4 g [8, 9] correlating well with Robert's study (1.3 g versus 4.9 g) [4]. Our case is unusual in that we report a benign adenoma with PTH, CCa^2+^, and weight that are all suggestive of parathyroid carcinoma.

There are several published cases where benign disease presents suspiciously and PTH in particular reaches levels associated with carcinoma. These include parathyroid cysts, those presenting in developing countries, patients with oxyphil adenomas, and those presenting, as demonstrated in our case, in hyperparathyroid crisis. Parathyroid cysts are rare neck lesions [10]. Case reports and series investigating these have reported a wide range of PTH levels, fluctuating from normal to 2250 ng/L [11] with overlapping clinical and biochemical presentations of parathyroid cysts with carcinoma [12], making Preoperative differentiation difficult but important as surgical management will differ. In developing countries, benign adenomas are reported to present with a similar biochemical profile to carcinoma. Agarwal et al. reported PTH levels in benign disease similar to that in cancerous disease [13], attributing this to the late presentation of the disease and nutritional deficiencies. Oxyphil adenomas have been suggested to cause 3% of all adenomas. Recent case reports have suggested markedly elevated PTH levels up to 1291 ng/L [14], although often there are no systemic features suggestive of patients in crises. 

Our case of parathyroid crisis is a further example of suspiciously elevated PTH levels, suggestive of parathyroid carcinoma. A recent study published in 2008 looking at just under 300 patients undergoing procedures for hyperparathyroidism showed 2.8% present in crisis [15]. These patients present with 3-4 times higher PTH levels than those with asymptomatic disease and over 10 times that of normal (up to 1770 ng/L), consistent with PTH levels in malignant disease. They found that 88% were caused by underlying adenoma. Bleeding into adenomas that have undergone cystic degeneration is a well-recognised cause of crisis with histopathological findings revealing a large adenoma with cystic spaces filled with haemorrhagic fluid [16]. There is a suggested trend where the weight of tumour excised during a hyperparathyroid crisis is heavier than those with asymptomatic disease (means 7.5 g versus 1.6 g) [15]. Our case is an example of this, where a patient in crisis has an adenoma weight much higher than typical benign disease.

Management of hyperparathyroid crisis has traditionally involved an emergency parathyroidectomy within 72 hours of presentation, which has a mortality of up to 14% [15, 17]. However, recent evidence published by Phitayakorn and McHenry [15]supports an early excision after a period of optimisation, rather than emergency surgery. Once volume depletion is corrected, loop diuretic therapy and intravenous bisphosphonate therapy can be initiated [18]. This relatively rapid reduction in CCa^2+^ thereby acts as a “bridge to surgery,” with a mean interval between presentation and operative intervention of 8 days [15]. This is not dissimilar to our interval of 10 days, thereby allowing for appropriate operative workup and management of concurrent medical problems. Surgical management of patients presenting with hyperparathyroid crisis secondary to adenomatous disease is effective, with a reported success rate of 92% [19]. While medical optimisation preoperatively is largely successful [15], there are reports where medical management has failed and disease has only responded to definitive surgical management [20]. Dialysis has also been used as a successful adjunct to medical therapy, in the interval between presentation and surgery [21]. With appropriate medical management prior to surgical excision, mortality rates have fallen, with rates for patients presenting in hyperparathyroid crisis reported as 2.8% [18].

In summary, our case involves a gentleman presenting in hyperparathyroid crisis with a massively raised serum PTH level that the authors believe to be one of the highest reported in benign disease published to date. It represents the presentation of benign cystic adenoma mimicking malignant disease. It also reinforces the importance of prompt initial medical management, Preoperative diagnostic and localising studies, and sound operative judgement, highlighting the difficulties facing the endocrine surgeon when dealing with lesions of the parathyroid gland.

## References

[1] Shane E (2001). Clinical review 122: parathyroid carcinoma. *Journal of Clinical Endocrinology and Metabolism*.

[2] Fernandez-Ranvier GG, Khanafshar E, Jensen K (2007). Parathyroid carcinoma, atypical parathyroid adenoma, or parathyromatosis?. *Cancer*.

[3] Ntaios G, Savopoulos C, Chatzinikolaou A, Kaiafa G, Hatzitolios A, Karamitsos D (2007). Parathyroid crisis as first manifestation of primary hyperparathyroidism. *European Journal of Internal Medicine*.

[4] Robert JH, Trombetti A, Garcia A (2005). Primary hyperparathyroidism: can parathyroid carcinoma be anticipated on clinical and biochemical grounds? Report of nine cases and review of the literature. *Annals of Surgical Oncology*.

[5] Hagag P, Kummer E, Weiss M (2008). Primary hyperparathyroidism: role of the preoperative oral calcium loading test in the differential diagnosis between adenoma and hyperplasia. *Calcified Tissue International*.

[6] Moretz WH, Watts TL, Virgin FW, Chin E, Gourin CG, Terris DJ (2007). Correlation of intraoperative parathyroid hormone levels with parathyroid gland size. *Laryngoscope*.

[7] Iwata T, Inoue K, Morita R (2008). Functional large parathyroid carcinoma extending into the superior mediastinum. *Annals of Thoracic and Cardiovascular Surgery*.

[8] DeLellis RA, Mazzaglia P, Mangray S (2008). Primary hyperparathyroidism: a current perspective. *Archives of Pathology and Laboratory Medicine*.

[9] Power C, Kavanagh D, Hill ADK, O’Higgins N, McDermott E (2005). Unusual presentation of a giant parathyroid adenoma: report of a case. *Surgery Today*.

[10] McKay GD, Ng TH, Morgan GJ, Chen RC (2007). Giant functioning parathyroid cyst presenting as a retrosternal goitre. *ANZ Journal of Surgery*.

[11] Ak I, Acikalin MF (2007). Hyperparathyroidism with a functioning parathyroid cyst. *Clinical Nuclear Medicine*.

[12] Wirowski D, Wicke C, Böhner H (2008). Presentation of 6 cases with parathyroid cysts and discussion of the literature. *Experimental and Clinical Endocrinology and Diabetes*.

[13] Agarwal G, Prasad KK, Kar DK, Krishnani N, Pandey R, Mishra SK (2006). Indian primary hyperparathyroidism patients with parathyroid carcinoma do not differ in clinicoinvestigative characteristics from those with benign parathyroid pathology. *World Journal of Surgery*.

[14] Dewanda NK, Chamber S, Tandon N, Karak AK (2000). Functioning oxyphil adenoma of parathyroid. *Journal of Postgraduate Medicine*.

[15] Phitayakorn R, McHenry CR (2008). Hyperparathyroid crisis: use of bisphosphonates as a bridge to parathyroidectomy. *Journal of the American College of Surgeons*.

[16] Manouras A, Toutouzas KG, Markogiannakis H (2008). Intracystic hemorrhage in a mediastinal cystic adenoma causing parathyrotoxic crisis. *Head and Neck*.

[17] Wang CA, Guyton SW (1979). Hyperparathyroid crisis. Clinical and pathologic studies of 14 patients. *Annals of Surgery*.

[18] Gasparri G, Camandona M, Mullineris B, Raggio E, Vigna S, Dei Poli M (2004). Acute hyperparathyroidism: our experience with 36 cases. *Annali italiani di chirurgia*.

[19] Cannon J, Lew JI, Solórzano CC (2010). Parathyroidectomy for hypercalcemic crisis: 40 years' experience and long-term outcomes. *Surgery*.

[20] Iihara M, Yamashita T, Tanaka R (1993). Seven patients with hyperparathyroid crisis: emphasis on prompt parathyroidectomy. *Nippon Naibunpi Gakkai Zasshi*.

[21] Georges CG, Guthoff M, Wehrmann M (2008). Hypercalcaemic crisis and acute renal failure due to primary hyperparathyroidism. *Deutsche Medizinische Wochenschrift*.

